# Model-Based Anticancer Effect of Botulinum Neurotoxin Type A1 on Syngeneic Melanoma Mice

**DOI:** 10.3389/fphar.2021.793349

**Published:** 2022-01-04

**Authors:** Won-Ho Kang, Hyo-Jeong Ryu, Seongsung Kwak, Hwi-Yeol Yun

**Affiliations:** ^1^ Department of Pharmacology and Toxicology, Gwangyo R&D Center, Medytox Inc., Suwon, South Korea; ^2^ Department of Pharmacy, College of Pharmacy, Chungnam National University, Deajon, South Korea

**Keywords:** botulinum neurotoxin, melanoma, pharmacokinetics, pharmacodynamics, K-PD modeling, tumor growth inhibition, NONMEM

## Abstract

In recent, Botulinum Neurotoxin A1 (BoNT/A1) has been suggested as a potential anticancer agent due to neuronal innervation in tumor cells. Although potential BoNT/A1’s mechanism of action for the tumor suppression has been gradually revealed so far, there were no reports to figure out the exposure-response relationships because of the difficulty of its quantitation in the biological matrix. The main objectives of this study were to measure the anticancer effect of BoNT/A1 using a syngeneic mouse model transplanted with melanoma cells (B16-F10) and developed a kinetic-pharmacodynamic (K-PD) model for quantitative exposure-response evaluation. To overcome the lack of exposure information, the K-PD model was implemented by the virtual pharmacokinetic compartment link to the pharmacodynamic compartment of Simeoni’s tumor growth inhibition model and evaluated using curve-fitting for the tumor growth-time profile after intratumoral injection of BoNT/A1. The final K-PD model was adequately explained for a pattern of tumor growth depending on represented exposure parameters and simulation studies were conducted to determine the optimal dose under various scenarios considering dose strength and frequency. The optimal dose range and regimen of ≥13.8 units kg^−1^ once a week or once every 3 days was predicted using the final model in B16-F10 syngeneic model and it was demonstrated with an extra *in-vivo* experiment. In conclusion, the K-PD model of BoNT/A1 was well developed to optimize the dosing regimen for evaluation of anticancer effect and this approach could be expandable to figure out quantitative interpretation of BoNT/A1’s efficacy in various xenograft and/or syngeneic models.

## Introduction

Botulinum neurotoxins (BoNTs) are generated by the anaerobic bacterium *Clostridium botulinum*. There are seven antigenically distinct botulinum toxin serotypes (A to G), of which BoNT type A1 (BoNT/A1) has traditionally been indicated for use in cosmetics and various diseases such as axillary hyperhidrosis, chronic migraine, and neurogenic detrusor overactivity (Nigam and Nigam, 2010; Chen, 2012; Walker and Dayan, 2014; Pirazzini et al., 2017; Kwak et al., 2020).

Owing to the increased understanding of the pharmacological importance of neovascularization and neuronal signaling in maintaining the tumor microenvironment there are several studies on the anticancer effect of BoNT/A1. It has been studied in various tumor types, including glioblastoma, neuroblastoma, prostate, breast, and colorectal cancer (Nam et al., 2012; Proietti et al., 2012; Ulloa et al., 2015; Rust et al., 2016; Mittal and Jabbari, 2020)**.** Additionally, several studies have shown that, similar to normal organs and tissues, solid tumors require innervation from the sympathetic and parasympathetic nervous systems to sustain the tumor microenvironment and facilitate cancer development, metastasis, and disease progression (Magnon et al., 2013; Coarfa et al., 2018; Faulkner et al., 2019; Reavis et al., 2020; Wang et al., 2020). Since BoNT/A1 causes denervation via cholinergic signaling pathway interference (Simpson, 2013; Kwak et al., 2020), exploring its anticancer properties is possible. In particular, various acetylcholine receptor subtypes occur in melanomas (Lucianò and Tata, 2020). More recently, it has been suggested that the presence of dorsal root ganglion neurons allows melanoma to grow significantly faster *in vivo*, and innervation plays a direct role in tumorigenesis by suppressing the immune response in melanoma (Keskinov et al., 2016; Reavis et al., 2020). Furthermore, a phase I clinical trial, including metastatic melanoma cancer patients, reported promising tumor inhibition activity administering a combination of pembrolizumab (PD-1 antibody) and propranolol, a non-selective beta-adrenergic blocking agent (Gandhi et al., 2021). Thus, we could conceptualize a relationship between tumor growth and innervation in melanoma. Additionally, melanoma can directly and precisely inject BoNT/A1 into the tumor site; therefore, we focused on it.

Although many studies have explored its anticancer effect and mechanism of action, quantitative exposure-response studies on BoNT/A1 have not been reported, as it is difficult to quantify the blood concentration of BoNT/A1 because of the extremely small volumes injected into the local tissue. Therefore, the regulatory agencies have approved BoNT/A1 products based on less in-depth PK data (Ravichandran et al., 2006; U.S. Food and Drug Administration, 2009; U.S. Food and Drug Administration, 2010; U.S. Food and Drug Administration, 2011). The lack of PK data is compounded by the difficulty of pharmacometrics research, including the prediction of exposure-response relationships.

**FIGURE 1:**
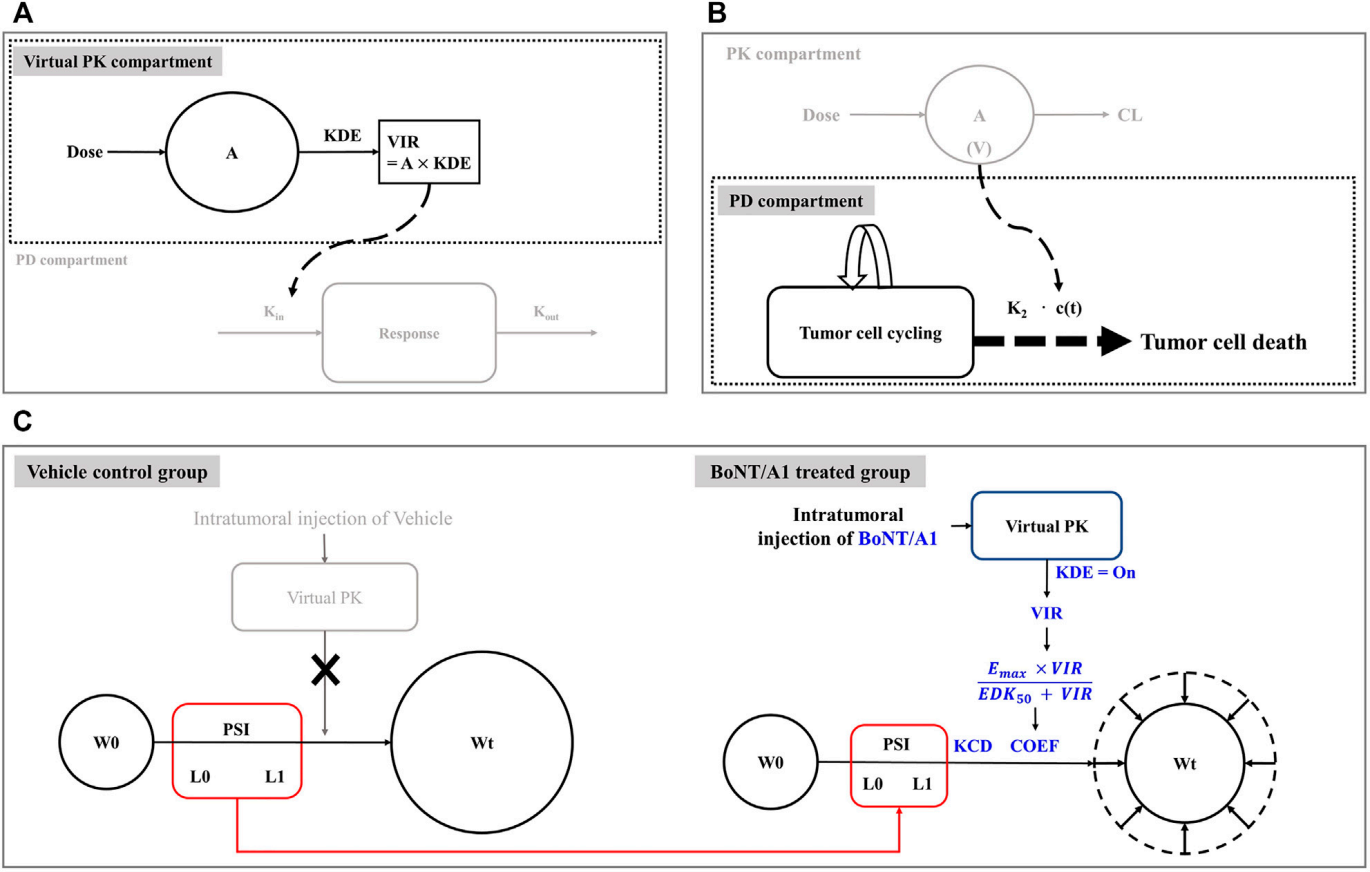
Model structures related to this study. **(A)** Typical K-PD model structure. **(B)** Simeoni’s tumor growth inhibition model structure. **(C)** Final K-PD model structure.

To overcome these issues, the kinetic-pharmacodynamic model (K-PD), also referred to as the kinetic drug action model, can provide an alternative. The K-PD model was established based on the link between virtual pharmacokinetic (PK) and pharmacodynamic (PD) compartments. The virtual PK compartment was reversely defined, depending on the curve fit for the PD compartment observation values (Jacqmin et al., 2007; Population Approach Group Europe, 2021; Pillai et al., 2004; González-Sales et al., 2017; Lixoft, 2021a, https://mlxtran.lixoft.com/examples/k-pd-models/). The typical K-PD model structure is shown in Figure 1A. The main advantage of the K-PD model is that quantitative analysis is possible even with a lack of PK information; however, pharmacometricians can only establish the exposure-response model to determine the optimal dosing regimen.

This study used a modified Simeoni tumor growth inhibition model as a PD model for our final model build-up and we set it up based on information from the Simeoni perturbed (treated group) and unperturbed (control group) model. In the unperturbed model, tumor growth was exponential in its early phase and linear in its late phase (Simeoni et al., 2004; Mo et al., 2014; Lixoft, 2021b, https://mlxtran.lixoft.com/model-libraries/tgi-library; Li et al., 2016). Eq. 1 provides a mathematical understanding of the overall tumor growth phenomenon, including exponential and linear growth. The perturbed model combined the drug effect on tumor growth with the unperturbed model. Here, the drug effect was revealed by the drug moving from the PK compartment, i.e., the intrinsic tumor growth aspect explained by the unperturbed model could be controlled by the inhibition ability of the drug flowing into the PD compartment. Eq. 2 provides a mathematical understanding of intrinsic tumor growth and drug inhibition.

$$\frac{dW\left(t\right)}{dt}=\frac{\lambda_{0}\cdot W\left(t\right)}{{\left[1\;+\;{\left(\frac{\lambda_{0}}{\lambda_{1}}\cdot W\left(t\right)\right)}^{\psi }\right]}^{1/\psi }}$$ (1)

$$\frac{dW\left(t\right)}{dt}=\frac{\lambda_{0}\cdot W\left(t\right)}{{\left[1\;+\;{\left(\frac{\lambda_{0}}{\lambda_{1}}\cdot W\left(t\right)\right)}^{\psi }\right]}^{1/\psi }}\;-k_{2}\;\cdot c\left(t\right)\cdot x\left(t\right)$$ (2)

where W(t) represents the tumor volume at time t, and λ0 and λ1 are exponential and linear growth rate constants, respectively. x(t) represents the proliferating portion of W(t). k_2_ is the tumor degradation rate constant. c(t) represents the drug concentration input from the PK compartment. The power coefficient Ψ (PSI) was assumed to be 20 as a switch between the exponential and linear tumor growth phases. In Simeoni’s experience, when the value was fixed 20, it was considered to be a sharp function as a switch (Simeoni et al., 2004). The original typical structure of Simeoni’s model was shown in Figure 1B.

The first objective of this study was to develop a novel tumor growth inhibition model to explain the anticancer effect of BoNT/A1 in a melanoma syngeneic mouse model. Second, to predict the relationship of exposure-response based on our developed K-PD model, and finally, to obtain a quantitatively appropriate therapeutic dose range and regimen for single- and multiple-dose studies. Furthermore, we considered that the K-PD modeling approaches will inform pharmacometricians on the feasibility of a K-PD model build-up for BoNT/A1.

## Materials and Methods

### Tumor Growth Inhibition in Syngeneic Melanoma Mice

Male C57BL/6 mice (six-week-old) were purchased from Orient Bio, Inc. (Seongnam-si, Republic of Korea) and kept in an environment with a 12-h light/dark cycle, controlled temperature (23 ± 3°C), relative humidity (55 ± 15%), and given free access to food and water. The B16-F10 (KCLB#80008) mouse melanoma cell line was obtained from the Korean Cell Line Bank (KCLB) and cultured in Dulbecco’s Modified Eagle Medium (11965-092, Gibco) supplemented with 10% fetal bovine serum (FBS, 10082-147, Gibco) and 1% penicillin and streptomycin (15140-122, Gibco) in a humidified atmosphere containing 5% CO_2_ at 37°C.

Thirty-five mice with tumor volumes above 25 mm^3^ were selected and assigned to one of five treatment groups, with seven mice per group. The mice were anesthetized with ketamine hydrochloride (100 mg kg^−1^) and xylazine (10 mg kg^−1^) by intraperitoneal injection prior to B16-F10 tumor cell implantation. Approximately 5 × 10^5^ B16-F10 cells in 1 ml phosphate-buffered saline were subcutaneously implanted into their right flank. The group assignment, performed 8 days post B16-F10 tumor implantations, was based on the tumor volume in the right flank of the tumor-bearing mice.

BoNT/A1 (CORETOX^®^ 100 U, Medytox Inc. Rep, Korea) was injected intratumorally on the day following the group assignment. The tumor volume at the inoculation site was determined by measuring the surface length and width of the tumor mass using a digital Vernier caliper (CD-15APX, Mitutoyo, Japan) and calculating according to Eq. 3 (Jensen et al., 2008; Faustino-Rocha et al., 2013; Pierrillas et al., 2016). The tumor volumes were measured and recorded twice a week (Table 1). All experimental procedures were approved by the Institutional Animal Care and Use Committee of Medytox Inc. (IACUC, Approval No. A-2020-004, January 29, 2020) before the initiation of the study.

$$Tumor\;volume\;\left(mm^{3}\right)=\frac{length\;\left(mm\right)\;\cdot \;width^{2}\left(mm^{2}\right)}{2}$$ (3)

**TABLE 1:** Study design for tumor growth inhibition using a syngeneic melanoma mouse model.

Group	Test articles	Dose (U kg^−1^)	Number of animals	Inoculation time (h) for tumor cell	Dosing route	Dosing time (h)	Measurement time (h)
G1	Vehicle	0	7	0	I.T.^a^	192	192, 264, 336, 432
G2	BoNT/A1	1.5	7				
G3	BoNT/A1	5	7				
G4	BoNT/A1	15	7				
G5	BoNT/A1	50	7				

a I.T.: intratumoral injection.

### Establishment of Tumor Growth Inhibition Model Using Virtual PK Concept

We established tumor inhibition models using a virtual PK compartment for mouse syngeneic melanoma models (Figure 1C). The modeling was performed using non-linear mixed-effects modeling (NONMEM) version 7.4 (ICON Development Solutions, Ellicott City, MD, United States), Pirania ver 2.9.8 (Princeton, NJ, United States), and Pearl-Speaks-non-linear mixed-effects modeling (PsN) ver 4.9.0 (Husargatan, Uppsala, Sweden). The statistical and graphical analyses were performed using R software ver. 3.6.1 (Welthandelsplatz, Vienna, Austria), R Studio ver. 1.2.1335, and GraphPad Prism ver. 7.05.

We combined the Simeoni tumor growth inhibition model with the virtual PK model to explain the tumor growth aspect after intratumoral injection of BoNT/A1 (Figure 1C). In the vehicle control group, we assumed that the growth of tumor cells followed the exponential (L0) and linear (L1) phases (Figure 1C). The growth rate constants L0 and L1 were assumed to be the same in the vehicle control and treated groups because the tumor growth rate is an intrinsic value for its growth. In the BoNT/A1-treated group, we assumed that BoNT/A1 from the virtual PK compartment inhibited intrinsic tumor growth, i.e., we hypothesized that the efficiencies and rates of tumor growth inhibition were generated by BoNT/A1 delivered through the virtual PK compartment. As mentioned in the introduction, in the K-PD model, all PK functions can be implicated by the virtual infusion rate instead of the typical PK parameters such as clearance, volume of distribution, absorption rate constant, and bioavailability, when the drug concentration data is unavailable.

Differential equations were used to describe the tumor growth pattern after intratumoral BoNT/A1 injection.

$$\frac{dA\left(1\right)}{dt}=-KDE\cdot A\left(1\right)$$ (4)

VIR =A(1)⋅KDE (5)

$$COEF=\;\frac{E_{max}\;\cdot \;VIR}{EDK_{50}\;+\;VIR}$$ (6)

$$\frac{dA\left(2\right)}{dt}=W0+\frac{L_{0}\cdot A\left(2\right)}{{\left[1\;+\;{\left(\frac{L_{0}}{L_{1}}\cdot A\left(2\right)\right)}^{\psi }\right]}^{1/\psi }}\;-KCD\;\cdot COEF\;\cdot \;A\left(2\right)$$ (7)

where A (1) and KDE represent the quantity of BoNT/A1 and degradation rate constant in the virtual PK compartment, respectively, and the virtual infusion rate (VIR) represents the virtual infusion rate from the virtual PK compartment to the PD compartment. W0 represents the tumor volume at the coefficient for the drug effect by describing the general Emax equation. KCD represents the cancer degradation rate constant. To summarize the function in the final K-PD model, when cancer cells grow by L0 and L1, BoNT/A1 flows into the PD compartment by VIR. Subsequently, BoNT/A1 within the PD compartment generated an inhibitory effect on cancer growth, defined by COEF and KCD (Figure 1C).

### Model Diagnostics and Evaluation

We checked the goodness-of-fit (GOF) plot of the final K-PD model vehicle and treated the model for two types of tumors. A visual predictive check (VPC) was also performed to evaluate the final K-PD model. Using the final K-PD model, 1,000 simulated replicates of the original dataset were generated, and the fifth percentile, median, and 95th percentile calculated from the simulated tumor volume were compared to the observed tumor volume. In addition, a bootstrap analysis was conducted to evaluate the internal model. The final K-PD model was compared with the 95% confidence intervals in the bootstrap analysis.

### Simulation Studies for Optimal Dosing Regimen

We conducted exploratory simulation studies based on the final K-PD model to establish an effective dose range and adequate dose regimen. First, the tumor growth-time profile was simulated for a single BoNT/A1 dose in the range of 0.0025–3,200 units kg^−1^ (U kg^−1^) to calculate the 50% effective dose (ED_50_), maximum effective dose (ED_max_), and the hill slope sigmoidity for the profiles. We then calculated the PD parameters using the sigmoid E_max_ model (Eq. 8) with tumor growth inhibition (TGI) for each dose. Consequently, we defined an adequate dose range using the ED_50_ value and maximum injectable dose (D_max-inj._) converted from the human dose (ClinicalTrials.gov., 2020) based on body surface area between species. That is, the D_max-inj_ was calculated using equation (Eq. 9) modified from the FDA guidelines relating to estimating the maximum safe starting dose in initial clinical trials (U.S. Food and Drug Administration. Center for Drug Evaluation and Research. U.S., 2005)

$$Effect=\;\frac{ED_{max}\;\times Dose^{\gamma }}{ED_{50}\;+\;Dose^{\gamma }}$$ (8)

$$Mouse\;D_{max-inj.}\;=Human\;D_{max-inj.}\;\times \;\frac{Human\;K_{m}\;\left(37\right)}{Mouse\;K_{m}\left(3\right)}$$ (9)

Second, we conducted a simulation study of the tumor growth pattern after multiple BoNT/A1 doses in various scenarios. We simulated three different dose regimens, once a day (Q.D.), once every 3 days (Q.3.D.), and once per week (Q.W.) in our study design. The dose range in the multiple-dose simulation study was 6–74 U kg^−1^ with ED_50_ and D_max-inj._ obtained from the single-dose simulation. The study designs are presented in detail in Table 2.

**TABLE 2:** Final melanoma syngeneic mouse K-PD model simulation scenarios and results.

Dosing frequency	Dose strength	Dose regimen	Dosing time (h)	Tumor growth inhibition^a^ (%)
Single	0	Q.D.^b^	192	0
	0.0025			14.92
	0.005			14.93
	0.02			15.01
	0.1			15.5
	1			20.63
	10			38.32
	50			53.47
	200			65.19
	400			69.6
	800			73.66
	1,600			76.94
	3,200			79.52
Multiple	6	Q.D.	192, 216, 240, 264, 288, 312, 336, 360, 384, 408, 432, 456	77.27
	13.8			81.17
	31.7			82.03
	74			82.91
	6	Q.3.D.^c^	192, 264, 336, 408	64.92
	13.8			74.78
	31.7			78.67
	74			81.53
	6	Q.W.^d^	192, 360	42.14
	13.8			59.61
	31.7			67.09
	74			74.13

B16-F10 tumor cell inoculation time was 0 h in every scenario. Administration route was intratumoral injection in every scenario.

a TGI values were calculated based on tumor volume at 468 h, when tumor volume of the vehicle control group >2000 mm^3^, standard for euthanasia.

b Q. D. once a day.

c Q. 3. D. once every 3 days.

d Q. W. once a week; TGI, tumor growth inhibition.

## Results

### Tumor Growth Profiles in Syngeneic Mice

**FIGURE 2:**
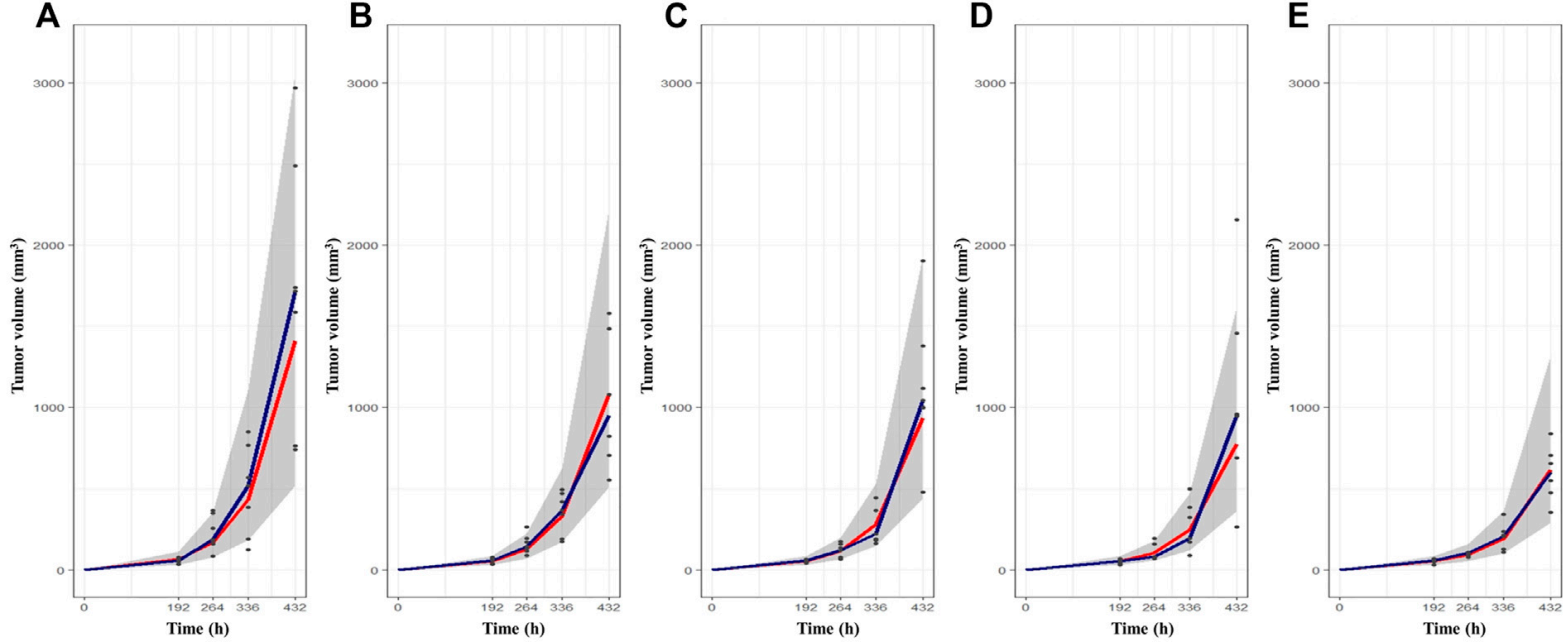
The tumor growth-time profiles after BoNT/A1 injection and visual check prediction (VPC) results for final K-PD model. **(A)** Vehicle control group. **(B)** BoNT/A1 1.5 U kg^−1^ treated group. **(C)** BoNT/A1 5 U BoNT/A1 1.5 U kg^−1^ treated group. **(D)** BoNT/A1 15 U kg^−1^ treated group **(E)** BoNT/A1 50 U kg^−1^ treated group. Black dot, observed tumor volume; Gray shade, 90% simulation intervals; Blue line, observed median value; Red line, simulated median value.

The change in tumor volume over time was determined from *in vivo* TGI experiments using syngeneic melanoma mouse models. The tumor growth patterns after BoNT/A1 injection are shown in Figure 2. Overall, the tumors grew slowly in the early stage and then rapidly when they exceeded a specific tumor volume. Figure 2A shows that the tumor growth pattern in each vehicle control group changed from an exponential to a linear function at a tumor volume of approximately 500 mm^3^. Additionally, a large magnitude of inter-individual variability within the data in the syngeneic melanoma mouse model was found; and at the last measurement, the coefficient of variation between the observations was approximately 47% in the vehicle control group. We used population PK-PD analyses in the model build-up to explain these magnitudes of inter-individual variability; furthermore, as the BoNT/A1 dose increased from 1.5 to 50 U kg^−1^, the tumor’s size decreased (Figures 2B–E). Note that, for all test groups, we designated the endpoint of tumor growth observation to be the time point (432 h) at which the tumor volume of the vehicle control group approached 2000 mm^3^ since this is the IACUC-recommended time point for euthanasia, considering animal ethics and welfare.

### Tumor Growth Inhibition Modeling

The dataset for the tumor growth pattern in the syngeneic melanoma mouse was applied to our novel TGI modeling system. Our final K-PD model best described the tumor volume from tumor volume-time profiles, which indicated a combination of the modified Simeoni TGI model with a virtual PK compartment. The estimated parameters are summarized in Table 3.

**TABLE 3:** Parameters estimated via final K-PD model and bootstrap validation.

Group	Parameter	Unit	Estimates (%RSE)	IIV (%RSE)	Bootstrap median (2.5–97.5% Percentile)
Vehicle group	L0	h^−1^	0.013 (8.9%)	13.5% (21.9%)	0.013 (0.011–0.021)
	L1	h^−1^	16.7 (15.6%)	—	16.4 (9.9–23.5)
	W0	mm^3^	0.0736 (14.9%)	—	0.0727 (0.0203–0.0965)
	Proportional error	N/A	0.285 (8.9%)		—
BoNT/A1 treated group	KDE	h^−1^	0.0292 (37.3%)	—	0.0278 (0.0098–0.0597)
	L0	h^−1^	0.013 FIX	8.8% (17.2%)	—
	L1	h^−1^	16.7 FIX	62.2% (43.9%)	—
	KCD	h^−1^	0.0427 (45.7%)	—	0.0442 (0.0360–2.2053)
	WO	mm^3^	0.0631 (4.3%)		0.0632 (0.0583–0.0683)
	EMAX	mol	0.164 (35.1%)		0.170 (0.138–8.470)
	EDK50	Unit/h	0.00116 (93.1%)		0.00127 (0.00016–24.8405)
	Proportional error	N/A	0.246 (6.9%)		—

IIV, inter-individual variability; RSE, relative standard error.

L0 and L1 were 0.013 and 16.7 h^−1^, respectively, in the B16-F10 syngeneic mouse model. The B16-F10 tumor cells grew rapidly during the linear growth phase following the exponential growth phase in the early stage of cancer cell transplantation. In the BoNT/A1-treated groups, the KDE was estimated at 0.0292 h^−1^ (low value) since BoNT/A1 was injected directly into the tumor tissue, the site of the TGI response. The E_max_ and EDK_50_ in the E_max_ formula reflecting the VIR [VIR = −KDE*A (1)] were 0.164 mol and 0.00116 U h^−1^, respectively. An EDK_50_ of 0.00116 U h^−1^ indicated that a small quantity of BoNT/A1 would be sufficient for B16-F10 growth inhibition because the EDK_50_ showed *in vivo* potency, defined as the infusion rate resulting in a 50% inhibition coefficient.

### Model Diagnostics and Evaluation

The VPCs with 95% prediction intervals using the final K-PD models are shown in Figure 2. The VPC plot indicated that most of the values were within the 95% prediction interval of the simulation data. The results indicated that the predictive performance was appropriate for the final K-PD models to explain TGI in the syngeneic melanoma model data. The values were similar to those generated from 1,000 bootstrap replications, indicating good precision in the final K-PD models (Table 3). The basic GOF plots for the final K-PD models are shown in Supplementary Figure S1. Individual and population predictions were evenly distributed across the line of identity, indicating good model fitting.

### Simulation Studies for Optimal Dosing Regimen

#### Simulation Study for Single BoNT/A1 Dose

**FIGURE 3:**
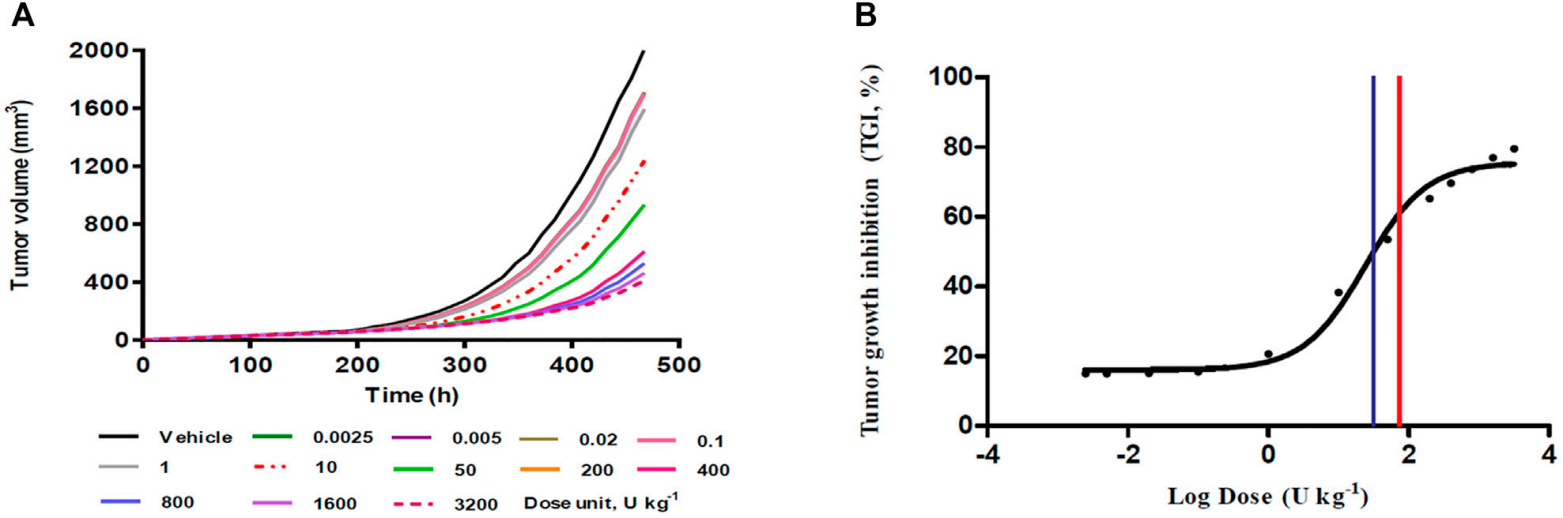
Single-dose simulation study. **(A)** Tumor growth-time profiles **(B)** Dose-response relationship from single-dose (%) simulation. Blue vertical line in the panel **(B)**, 31.7 U kg^−1^ as a predicted ED_50_; red vertical line in the panel **(B)**, 74 U kg^−1^ as a predicted maximum injectable dose (D_max-inj._).

The tumor growth-time profiles were simulated at single BoNT/A1 doses ranging from 0.0025 to 3200 U kg^−1^ using our novel K-PD model. In all groups, exponential growth was observed after tumor transplantation and linear growth toward the late stage. In addition, an increase in tumor suppression was observed with an increasing BoNT/A1 dosage (Figure 3A). The TGI rate of each BoNT/A1 treatment group was calculated based on 468 h, when the tumor volume of the vehicle group exceeded 2000 mm^3^ (Table 2). The dose-response relationship estimated with a typical sigmoidal E_max_ equation had an ED_50_ of 31.7 U kg^−1^. The mouse D_max-inj._ was 74 U kg^−1^ calculated from human D_max-inj._ (ClinicalTrials.gov. 2020) at 6 U kg^−1^ (360 U) using an inter-species conversion factor on the basis of body surface area (U.S. Food and Drug Administration. Center for Drug Evaluation and Research. U.S., 2005). Consequently, we identified the dose range of 31.7–74 U kg^−1^ as a therapeutically meaningful range for the BoNT/A1 single-injection study (Figure 3B).

#### Simulation Study for Multiple BoNT/A1 Doses

**FIGURE 4:**
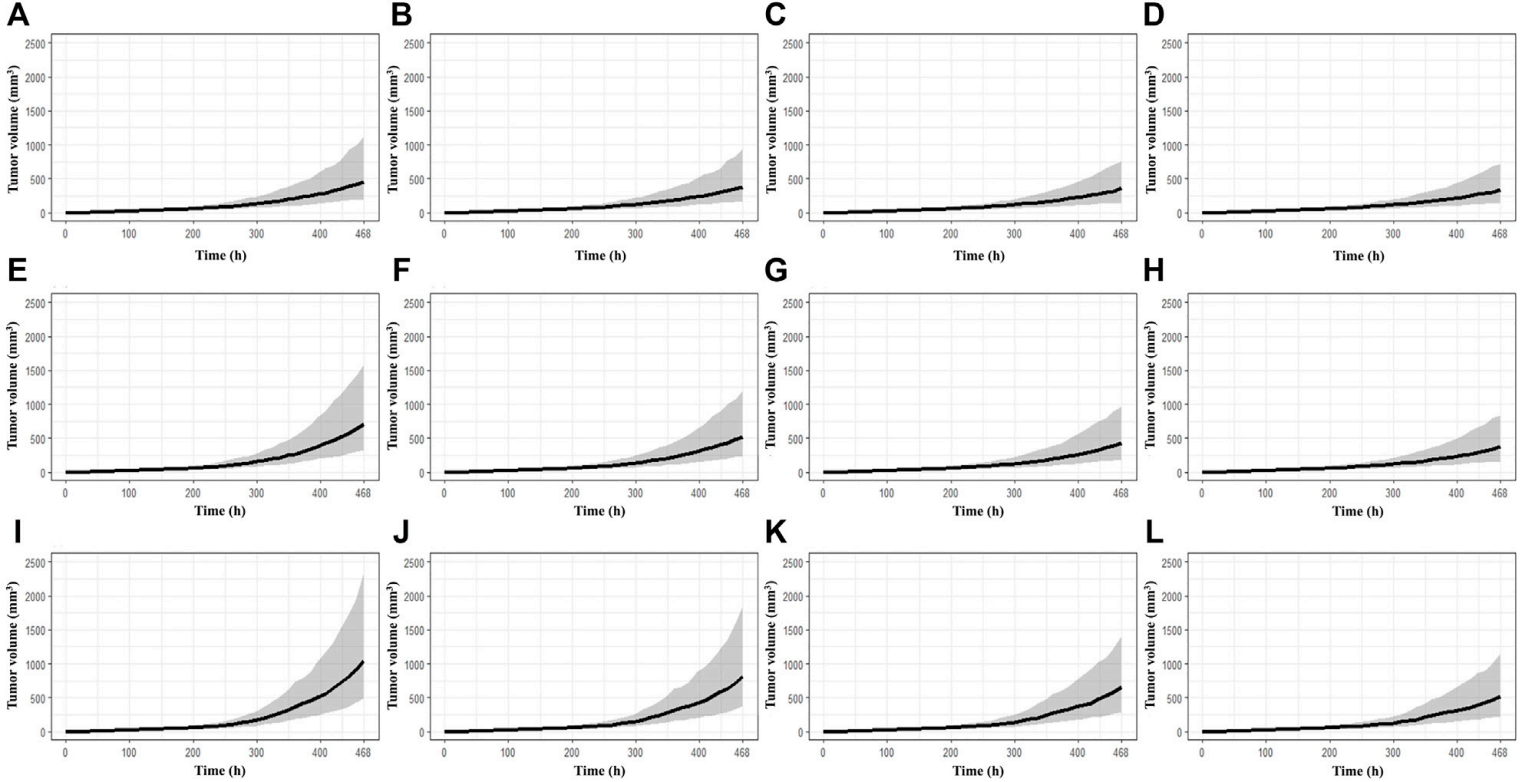
Multiple-dose simulation study. **(A)** BoNT/A1 6 U kg^−1^ for Q.D. **(B)** BoNT/A1 13.8 U kg^−1^ for Q.D. **(C)** BoNT/A1 31.7 U kg^−1^ for Q.D. **(D)** BoNT/A1 74 U kg^−1^ for Q.D. **(E)** BoNT/A1 6 U kg^−1^ for Q.3.D. **(F)** BoNT/A1 13.8 U kg^−1^ for Q.3.D. **(G)** BoNT/A1 31.7 U kg^−1^ for Q.3.D. **(H)** BoNT/A1 74 U kg^−1^ for Q.3.D. **(I)** BoNT/A1 6 U kg^−1^ for Q.W. **(J)** BoNT/A1 13.8 U kg^−1^ for Q.W. **(K)** BoNT/A1 31.7 U kg^−1^ for Q.W. **(L)** BoNT/A1 74 U kg^−1^ for Q.W.

**FIGURE 5:**
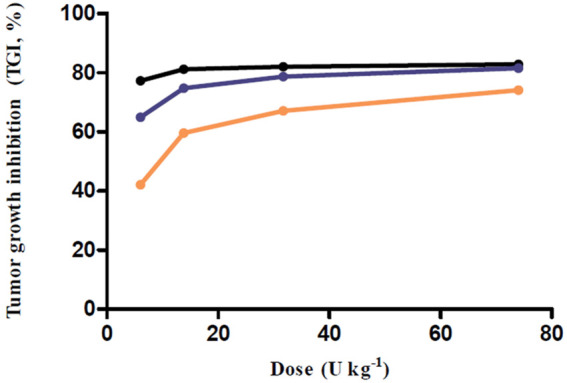
Dose-response relationship for multiple-dose simulations. Black line, Q.D. as a dose regimen; Blue line, Q.3.D. as a dose regimen; Orange line, Q.W. as a dose regimen; Every closed circle, observed TGI values.

The simulations at four different dose regimens, including ED_50_ and D_max-inj._ in three-dose regimen conditions, revealed the results shown in Figure 4 and Table 2. First, when the BoNT/A1 dose was increased from 6 to 74 U kg^−1^ in the Q.D. dose regimen scenarios, the TGI was 77.27–82.92%, and a significant anticancer effect was observed at all doses (Figures 4A–D). Second, when the dose was increased from 6 to 74 U kg^−1^ in the Q.3.D. scenarios, the TGI (%) value was 64.92–81.53%, and there was a meaningful anticancer effect depending on the dose escalation in those scenarios (Figures 4E–H). Finally, the TGI of Q.W. was 42.14–74.14% at the same dose range in previous studies. These results indicated a good dose-dependent escalation in anticancer effect (Figures 4I–L). The dose-response relationships for the three different dose regimens are shown in Figure 5. In the Q.D. dose regimen scenario, a high TGI of ≥77% was maintained at all dose ranges. For the Q.3.D. dose regimen, a high TGI of ≥74% was observed in all dose ranges except at 6 U kg^−1^, which was 64%. Q.W. the last dose regimen scenario among our repeated simulation studies, showed a significant logarithmic TGI increase of between 6 and 74 U kg^−1^.

## Discussion

As with BoNT/A1, because the plasma drug-time-concentration profile might not always be available to correlate exposure with the biomarker or clinical endpoints during the drug discovery and development process, pharmacometricians needed to examine alternatives for cases in which PK data are unavailable. Using the syngeneic melanoma mouse model, we attempted to establish a mathematical model to explain the anticancer effect in a diseased mouse model when BoNT/A1 was intratumorally injected in a situation where PK data were not available. We attempted to combine the virtual PK part of the K-PD model with the PD part of the Simeoni TGI model. Our final K-PD model predicted the anticancer effects well. First, our final K-PD model estimated that the tumor growth rates L0 and L1 were fast for the dataset of the syngeneic melanoma mice. In addition, the virtual PK parameters were also well and stably estimated while performing curve-fitting using only the tumor volume as a dependent variable. While performing NONMEM iterations, the virtual PK parameters were also stably estimated. Meanwhile, we considered L0 and L1 omega in the final K-PD model to account for the large inter-individual variability (IIV) in tumor growth profiles (Figure 2). In developing the model, the IIV for the other parameters was considered, but it had little effect on decreasing the objective function value. Therefore, we only reflected IIV on L0 and L1 in our final K-PD model.

Based on the established K-PD model, we simulated various scenarios. First, a simulation study of a single BoNT/A1 administration was conducted. Due to the BoNT neurotoxicity in this simulation study, the dose was gradually increased to a high dose (0.0025–3200 U kg^−1^) that could not be injected in an actual experiment, thereby securing a dose-response profile for the anti-melanoma effect—using the calculated ED_50_ and the mouse D_max-inj._ converted from the human, D_max-inj._ we confirmed that the therapeutic dose range of BoNT/A1 in syngeneic melanoma mice was approximately 31.7–74 U kg^−1^ (Figure 3; Table 2). Second, we conducted another simulation study on multiple BoNT/A1 doses from 6 to 74 U kg^−1^ in three-dose regimens (Q.D. Q.3.D., and Q.W.). The results confirmed the dose-proportional and superior anticancer effect in the all-dose regimen scenarios (Figure 5; Table 2). Overall, we considered that the minimum meaningful dose in syngeneic melanoma mice is ≥13.8 U kg^−1^ (0.27 U). We considered that the adequate administration interval could be Q.3.D. or Q.W. In the case of Q.D. there could be a neurotoxicological concern due to the injection of dense BoNT/A1. Meanwhile, in some BoNT/A1 products such as Dysport^®^ (Ipsen, United States), there was a precedent in which repeated toxicity tests were conducted by once-daily intramuscular administration for 14 days in rats, but the No Observed Adverse Effect Level (NOAEL) in this study was low such as 1 U Rat^−1^ (∼5 U kg^−1^) (U.S. Food and Drug Administration, 2009). Therefore, there were still concerns concerning neurotoxicity in Q.D. even though there were differences in animal species and administration sites in the tests. Furthermore, our results indicated that BoNT/A1 alone had a highly significant anticancer effect; therefore, it could be difficult to confirm the synergistic effect with other anticancer drugs such as PD-1 or PD-L1 antibody.

Fortunately, the predicted optimal dosing regimen was verified through additional *in-vivo* results in the B16-F10 syngeneic mouse model by performing an *in vivo* anticancer efficacy study with single or multiple intratumoral BoNT/A1 injections. The anticancer efficacy study conducted using BoNT/A1 injections at three doses (Supplementary Table S1) showed that most tumor volumes were within the 90% simulation interval estimated using our model (Supplementary Figure S2). Furthermore, when we compared the average observed values at 432 h, the final measurement time, with the simulation median value and interval, we found that all average observation values were within the 90% prediction interval (Supplementary Table S2). Based on the above results, we again found that our final K-PD model had appropriate predictive power. Furthermore, we applied our K-PD model to the TGI data obtained from another syngeneic model with inoculated 4T-1 breast cancer cells and validated the model, as it explained the tumor growth aspect accurately. Even when BoNT/A1’s inhibitory effect in the 4T-1 syngeneic model was slightly lower than that of B16-F10, our K-PD model exhibited a good curve-fitting for the 4T-1 tumor volume-time curve (in-house results). In addition, in a syngeneic model inoculated with MBT2 cells, which are ovarian cancer cells, the tumor growth rates, such as L1 and L0, were high and similar to that of B16-F10. Hence, for such tumor cell line types, we presume that growth rate constants similar to that of B16-F10 can be used (in-house results).

A previous study had identified the BoNT/A1 antitumoral effect in prostate cancer via the mouse xenograft experiment and clinical trials (Coarfa et al., 2018). As the result of the study, there was a reduction of tumor incidence and size where BoNT/A1’s dose was 0.45 U *via* intratumoral injection route to the xenograft mouse and there were meaningful findings which were not only nerve density decrease but apoptosis increases in prostate cancer when 100 U of BoNT/A1 was treated to the patients in clinical trials. Although our targeted cancer is different from this previous study, we considered that the results could be indirectly applied to our case because we have obtained a similar efficacious dose of BoNT/A1. In detail, we could expect a similar or lower effective dose (≤100 U) in the clinical study for melanoma patients considering the effective dose (100 U) of BoNT/A1 for prostate cancer patients since the minimum efficacious dose (0.27 U) in the syngeneic melanoma mouse was similar or a little lower than 0.45 U in the xenograft prostate mouse.

We are currently trying to expand the indications of BoNT/A1 by targeting melanoma that can be locally injectable. Although BoNT/A1 rarely elicits adverse events such as facial and palpebral edema, injection site pain, eye pain, erythema, psoriasis, skin infections, vertigo, nausea, fever, blepharitis, xerostomia, itching, and asthenia, we speculate that the minimum efficacious dose (0.27 U = 13.8 U Kg^−1^ ≈ 1.1 U Kg^−1^ for human) that we arrived at could be used for melanoma treatment in clinical trials considering the risk-to-benefit aspect.

There were some potential limitations in this study. First, our concept for the model development was applied only to B16-F10 melanoma tumors. Hence, it was unclear whether our concept for the combination model build-up could be applied to other tumors. Second, in this study, we used only tumor volume as a PD marker. However, if we had obtained the survival rate or the level of other biomarkers related to cancer growth, we would have succeeded in developing better models, such as disease progression models. Since a study to obtain these factors is planned, in this study, we focused on the development of models that could satisfactorily explain the change in tumor volume. Third, regarding the pharmacological mechanism of the anticancer effect of BoNT/A1, there have been various reports of BoNT/A1’s effects on apoptosis, phosphorylation process, and neuron denervation (Proietti et al., 2012; Bandala et al., 2013; Coarfa et al., 2018). Therefore, we have planned on performing *in-vitro* studies clarifying the pharmacological mechanism behind the anticancer effect. We believe that this investigation on the mechanism of tumor treatment could facilitate the development of more elaborate pharmacometrics models.

## Conclusion

We developed a mathematical model to explain the tumor-suppressing effect of BoNT/A1 in B16-F10 melanoma cancer cells. The virtual PK compartment of the K-PD model was used as the model setup for the PK part in a situation where PK data were not available, and the PD part of the Simeoni TGI model was used to explain the tumor growth aspect in our dataset. Hence, novel tumor growth inhibition models for BoNT/A1 were established by combining the PK and PD parts of each model. Furthermore, our final K-PD models were well curve-fitted for each animal experiment data set and showed adequate predictive power for both single and repeated BoNT/A1 injection data. Moreover, we hypothesize that this approach could be scalable for pharmacometricians to use in PK-PD modeling for BoNT/A1.

## Data Availability

The original contributions presented in the study are included in the article/Supplementary Material, further inquiries can be directed to the corresponding author.
