# Supplementary material for: Model-Based Anticancer Effect of Botulinum Neurotoxin Type A1 on Syngeneic Melanoma Mice
Source: Front Pharmacol. 2022 Jan 4;12:793349. doi: 10.3389/fphar.2021.793349 (PMC8763961; doi:10.3389/fphar.2021.793349)
Supplement: Supplementary file 1 [file DataSheet1.docx]

Supplementary Material

# Supplementary Figures and Tables

## Supplementary Figures


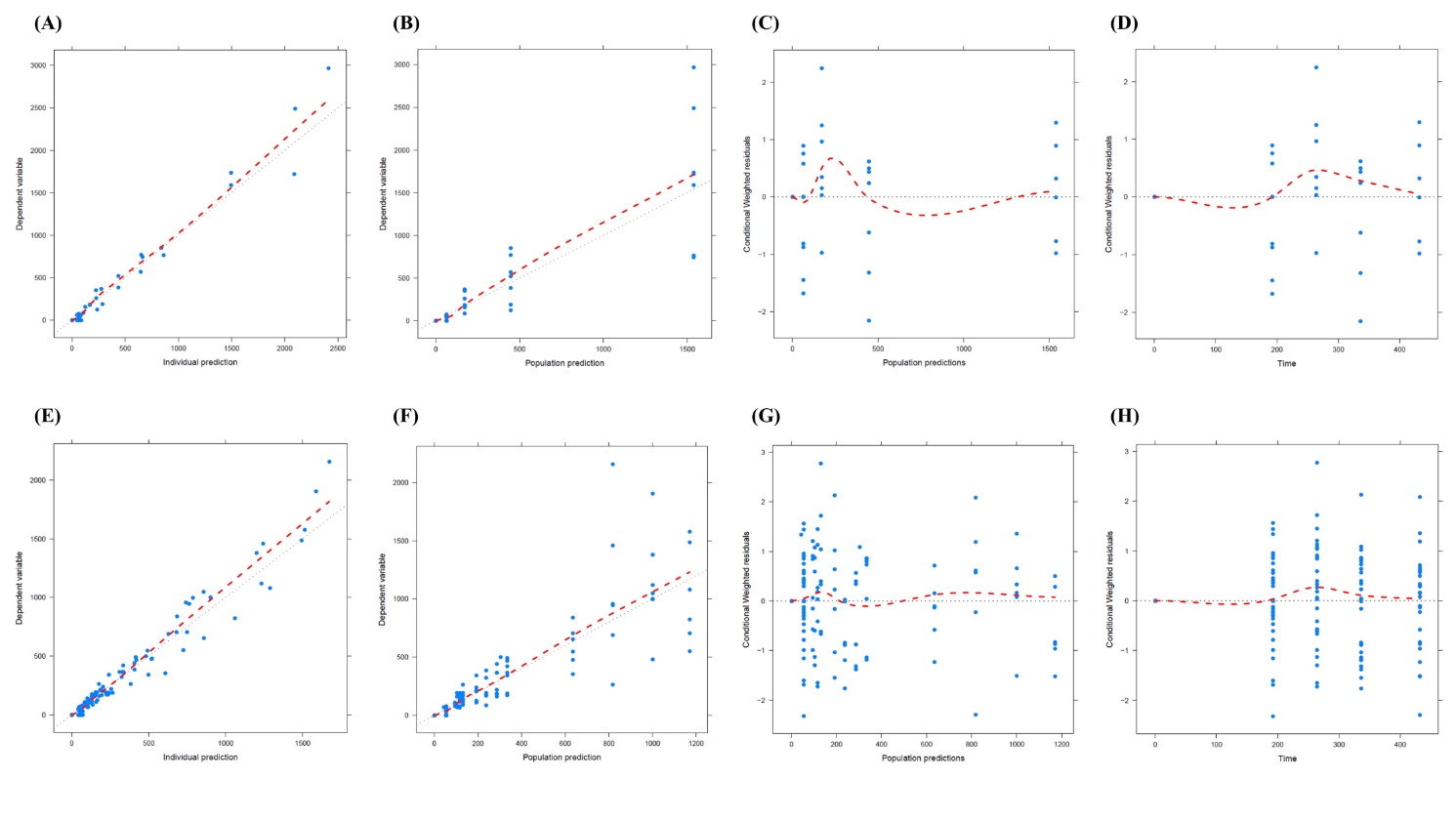


**Supplementary Figure S1.** The result of goodness of fit plot (GOF) for the final model. (A) Individual prediction versus dependent variables for vehicle group. (B) Population prediction versus dependent variables for vehicle group. (C) Conditional weighted residual errors versus population predictions for vehicle group. (D) Conditional weighted residual errors versus time for vehicle group. (E) Individual prediction versus dependent variables for treated group. (F) Population prediction versus dependent variables for treated group. (G) Conditional weighted residual errors versus population predictions for treated group. (H) Conditional weighted residual errors versus time for treated group.


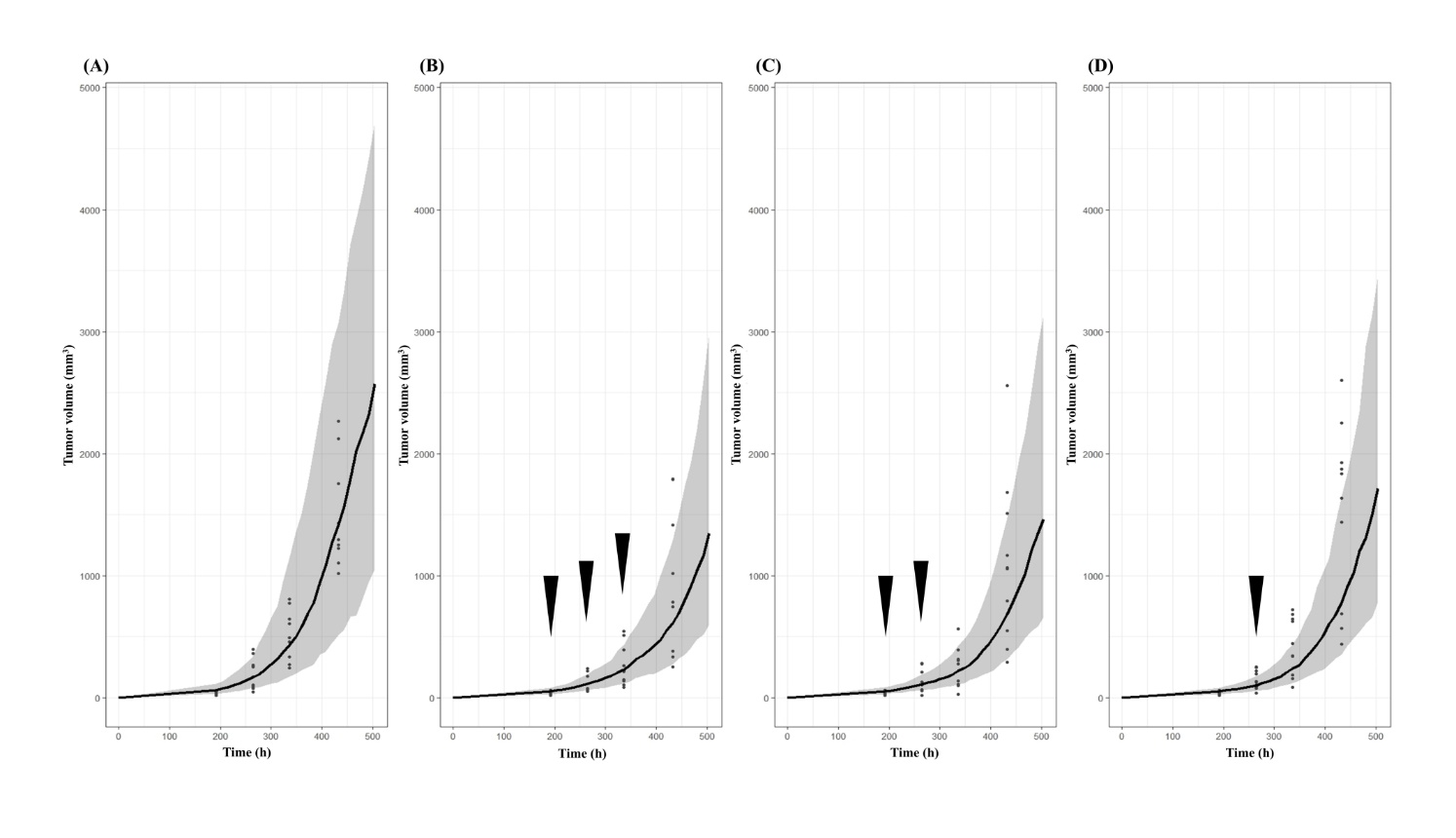


**Supplementary Figure S2.** The tumor growth-time profiles for additional animal experiment and the results of visual check prediction (VPC) results for the final model. (A) Group 1: Vehicle control group. (B) Group 2: Treated group for BoNT/A1 5 U/Kg (3 injection as a dose frequency). (C) Treated group for BoNT/A1 7.5 U/Kg (2 injection as a dose frequency). (D) Treated group for BoNT/A1 15 U/Kg (1 injection as a dose frequency). Black dot, observed tumor volume; Gray shade, 90% simulation intervals; Blacked line, simulated median value; Black reverse triangle, injection time for the BoNT/A1.

## Supplementary Tables

**Supplementary Table S1.** The additional study design using syngeneic melanoma mouse model.

| Group | Test articles | | Dose (U/Kg) | Number of animals | Inoculation time (hr) for tumor cell | Dosing route | Dosing time (hr) | Measurem-ent time (hr) |
| --- | --- | --- | --- | --- | --- | --- | --- | --- |
| G1 | | Vehicle | 0 | 10 | 0 | I.T.* | 192, 264, 336 | 192, 264, 336, 432 |
| G2 | | BoNT/A1 | 5 | 10 |  |  | 192, 264, 336 |  |
| G3 | | BoNT/A1 | 7.5 | 10 |  |  | 192, 264 |  |
| G4 | | BoNT/A1 | 15 | 10 |  |  | 192 |  |

*I.T., Intra-tumoral injection

**Supplementary Table S2.** The comparison between simulation and observation values at the additional study in syngeneic melanoma mouse model.

| Group | Observed average value  (mm^3^, @432 hr) | | Predicted values (mm^3^, @ 432 hr) | | | | |
| --- | --- | --- | --- | --- | --- | --- | --- |
|  |  |  | 5% quantiles | | median | | 95% quantiles |
| G1 | 1497.52 | 509.90 | | 1414.05 | | 3069.17 | |
| G2 | 946.23 | 271.25 | | 609.99 | | 1289.88 | |
| G3 | 1107.51 | 317.03 | | 683.21 | | 1472.03 | |
| G4 | 1526.10 | 354.24 | | 781.18 | | 1651.19 | |

G1, The group for vehicle control; G2, The group for 5 U/Kg of BoNT/A1; G3, The group for 7.5 U/Kg of BoNT/A1; G4, The group for 15 U/Kg of BoNT/A1.
